# *Trypanosoma cruzi* discrete typing unit TcIV implicated in a case of acute Chagas disease in a domiciliated dog in the western Amazon

**DOI:** 10.1590/0037-8682-0873-2020

**Published:** 2021-03-22

**Authors:** Luciene Almeida Siqueira de Vasconcelos, Josué Costa Oliveira, Rubens Celso Andrade da Silva, Silvia Cássia Brandão Justiniano, Éder dos Santos Souza, Laylah Kelre Costa Magalhães, Henrique Silveira, George Allan Villarouco da Silva, Jorge Augusto de Oliveira Guerra, Maria das Graças Vale Barbosa Guerra

**Affiliations:** 1 Fundação de Medicina Tropical Doutor Heitor Vieira Dourado, Centro de Entomologia, Manaus, AM, Brasil.; 2 Universidade do Estado do Amazonas, Programa de Pós-graduação em Medicina Tropical, Manaus, AM, Brasil.; 3 Universidade Nova de Lisboa, Instituto de Higiene e Medicina Tropical, Lisboa, Portugal.

**Keywords:** Trypanosomatidae, Surveillance, Domestic dog

## Abstract

Chagas disease is caused by the protozoan *Trypanosoma cruzi.* Seven lineages have been identified based on different molecular markers, namely TcI, TcII, TcIII, TcIV, TcV, TcVI, and TcBat. Dogs play the role of epidemiological sentinels being domestic reservoirs of *T. cruzi.* The aim of the current study was to report the first case of CD in a domestic dog in Manaus, Amazonas, Brazil, infected with *T. cruzi* DTU TcIV. We hope our report encourages veterinarians and surveillance professionals to a take a deeper look at *T. cruzi* infection in domestic animals.

## INTRODUCTION

*Trypanosoma cruzi* has biological plasticity that results in complex and peculiar transmission cycles in nature. It is classified into seven discrete typing units based on different molecular markers, namely TcI, TcII, TcIII, TcIV, TcV, TcVI, and Tc Bat^1^. Dogs play an important role in domestic transmission cycles of *Trypanosoma cruzi* and are considered a risk factor for human infection^2^. When dogs get infected, clinical signs of an acute phase and a chronic phase of the disease are detected as in humans^3^. 

Dogs generally show nonspecific clinical signs in the acute phase of this disease; however, the chronic phase is characterized by anorexia, fever, lymphadenopathy, cardiomyopathy, and right heart insufficiency. This clinical presentation is variable and depends on the type of strain involved in the infection, route of infection, and parasitic burden^4^.

For specific treatment of Chagas disease in dogs, two drugs are currently recommended: nifurtimox - 2 to 7 mg/kg orally every 6 hours for 3 to 5 months and benznidazole at a dose of 7 mg/kg orally every 12 hours for 2 months^5^. The aim of the current study was to report the first case of Chagas disease in a domestic animal in the city of Manaus, Amazonas, Brazil, infected with DTU TcIV lineage of *Trypanosoma cruzi,* with manifestations of acute disease. 

## CASE REPORT

A Golden Retriever dog, female, approximately 7 years old, weighing 15 kg, was admitted to a veterinary clinic in the city of Manaus, Amazonas, with complaints of apathy, loss of appetite, progressive weight loss, and dyspnea. The animal came from a private property located 41 km from the urban area of Manaus. Physical and laboratory examinations were performed. After receiving the results, new samples were collected and sent to the Heitor Vieira Dourado-FMT-HVD Tropical Medicine Foundation for further investigations including fresh examination, blood culture, xenodiagnosis, and Polymerase Chain Reaction PCR. 

For the fresh examination, 10µL of blood was placed on a slide under a 40x optical microscope to observe flagellate forms identical to *T. cruzi*. Five mL of blood was collected in a heparinized tube and for blood culture 100µl was inoculated into 2.5 mL of NNN culture medium containing rabbit blood and 40 mg/mL of gentamycin sulfate. 

For the extraction of *T. cruzi* DNA, 200µL of blood was processed using the protocol recommended by the manufacturer of the PureLink^TM^ Genomic DNA kit (Invitrogen by Thermo Fisher Scientific, Carlsbad, California, USA) and PCR, amplifying the glucose-phosphate isomerase (GPI) gene, primers gpi.for (59CGCACACTGGCCCTATTATT); gpi.rev (59TTCCATTGCTTTCCATGTCA) according to the manufacturer’s protocol^6^. The PCR product was purified using polyethylene glycol (PEG). For sequencing, the Big Dye Terminator^TM^ 3.1 kit (Applied Biosystems, Invitrogen, Bradford, Massachusetts, USA) was used, following the manufacturer's recommendations. The sample was processed in Applied Biosystems® 3130/3130xl Genetic Analyzers, validated, and paired with the reference strings deposited in GenBank. The *T. cruzi* DTU TcIV strain was detected.

Ethical aspects: The ethical aspects of this case report was submitted and aproved by the Ethics Committee on the use of animals of the Tropical Medicine Foundation Dr. Heitor Vieira Dourado.

## DISCUSSION

**FIGURE 1:**
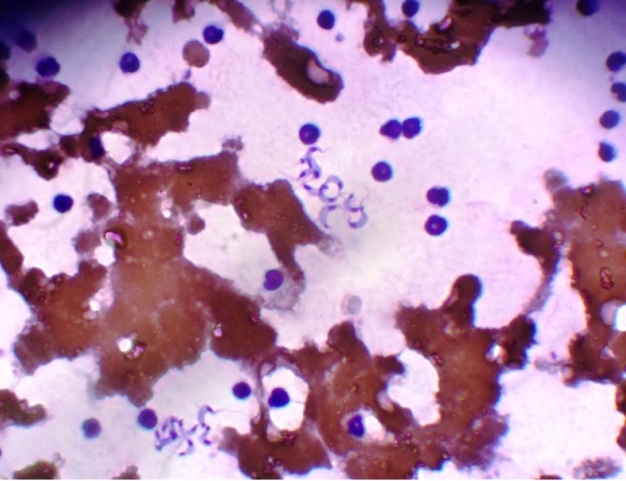
Fresh blood Smear showing flagellated forms of *Trypanosoma cruzi.*

During physical examination were observed: ocular and gingival mucous membranes pale, splenomegaly, hepatomegaly, generalized lymphadenomegaly, rectal temperature of 40.1 °C, respiratory rate of 48 Breathing Movements per minute(bm/min), and heart rate of 180 bpm. A Complete Blood Count (CBC) and blood smear were performed, revealing leukocytosis (27,300 mm³), neutrophilia (16380 mm³), eosinopenia (0 mm³), and monocytosis (4095 mm³) with 90% of monocytes showing active phagocytic activity. The presence of trypomastigote forms of *Trypanosoma* sp. on wet mount was observed. After six days, flagellate forms were seen on the wet mount. In the xenodiagnostic test, 20 *Rhodnius robustus* nymphs of the 3^rd^ instar were inoculated into the left flank region of the dog after trichotomy; the procedure was performed under a microscope. Ten days after the inoculation, triatomine feces were positive for the presence of flagellate forms (Figure 1). Thoracic radiography (cardiac evaluation), electrocardiogram, and echocardiogram were performed, as well as renal and liver function examinations, in order to complement the initial animal evaluation. Therapeutic treatment was initiated promptly with a dose of 7 mg/kg of benznidazole every 12 h, with an initial stipulated treatment of 2 months. However, 6 days after initiating the treatment, the animal died at the owner's home, and the owner did not authorize the post-mortem evaluation of the animal.

In the Amazon, records of cases of Chagas disease refer to the detection of human cases of Chagas disease mainly in the acute phase, through the malaria surveillance service, diagnosed using the thick drop test and blood smear^7^. Early registration of cases has contributed to low human mortality in this region^8^. In our case, according to the history received in the anamnesis, the animal lived on a private property located in a rural area on the outskirts of the peri-urban zone of Manaus, surrounded by a primary forest. These conditions would have favored the contact of the animal with an insect vector and consequently, with the parasite. The owner had been absent for several days, and when she returned to the site, she found the animal sick, and no one had noticed. It is likely that the time between the onset of the infection and the attendance by the veterinary health service was an important factor for the final outcome, considering that although the animal was taken for a veterinary consultation, the owner was unable to report how long the animal had been sick.

Although there is no colonization of triatomine vectors in households, there are regular records of accidental invasions of insects into homes or apartments built in areas close to forest fragments, both in rural and urban areas, indicating the vulnerability of the human population and their pets to come in contact with the parasite's wild cycle. In this context, in similar situations, parasitological diagnosis, whether direct (thick drop test) or indirect (xenodiagnosis), is essential for the initiation of adequate therapy at an early stage.

This vulnerability of domestic animals to infection by *T. cruzi* suggests the presence of infected dogs as a potential risk factor in the transmission of *T. cruzi* to humans. This case serves as a warning to the veterinary community to identify situations epidemiology comparable to that reported herein, as mathematical modeling has been shown to reinforce that dogs amplify domestic parasite transmission^9^. Dogs, as well as other domestic animals, can be infected by *Trypanosoma* spp. The occurrence of *T. cruzi-*infected animals in urban and rural areas provides epidemiological data for monitoring trypanosomatid infections^10^ ^-^ ^13^.

In this context, the need for guidance was observed, both among veterinary professionals and, mainly among the population, for a more sensitive examination of the possibility of *T. cruzi* infection in domestic animals, living in conditions similar to the reported case, considering that diagnosis in majority of the cases is limited by several factors including access to veterinary services.

**FIGURE 2:**
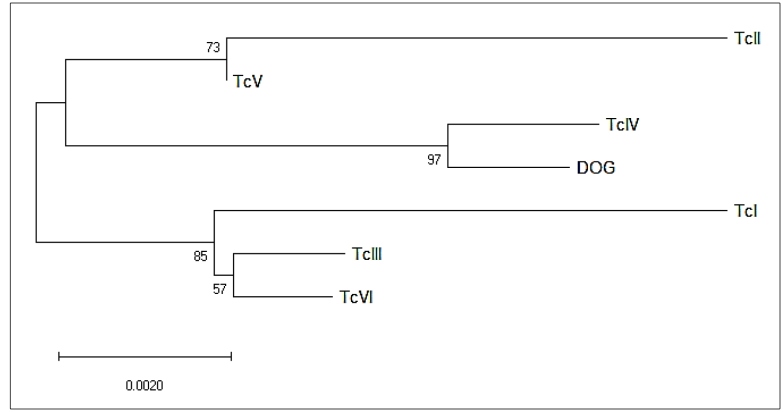
Phylogenetic reconstruction of a *T. cruzi* strain obtained from a dog in Manaus, Amazonas.

This report represents the first record of Chagas disease with a fatal outcome in a domestic animal infected with *T. cruzi* DTU TcIV (Figure 2), demonstrating that in the Amazon, where there is still no domiciliation of vectors, there is the possibility of contact between the population and their pets with the disease because of the proximity to the wild cycle of the parasite. This DTU TcIV has been found in humans, especially in patients with acute disease resulting from oral transmission^14^. There is an urgent need for guidance and awareness in the community, especially among residents of areas close to the wild environment, regarding the possibility of similar cases, especially in dogs, because of the proximity and the risk of contact with infected triatomines, considering that these animals have greater mobility between environments, making them vulnerable to the imminent risks of *T. cruzi* infection.
